# Supplementary material for: A Global Screen for Assembly State Changes of the Mitotic Proteome by SEC-SWATH-MS
Source: Cell Syst. 2020 Feb 26;10(2):133–155.e6. doi: 10.1016/j.cels.2020.01.001 (PMC7042714; doi:10.1016/j.cels.2020.01.001)

Q9H223 | EHD4_HUMAN | EHD4 HCA10 HCA11 PAST4 FKSG7
Monomer MW [kDa]: 61.175 Monomer expected elution fraction: 44

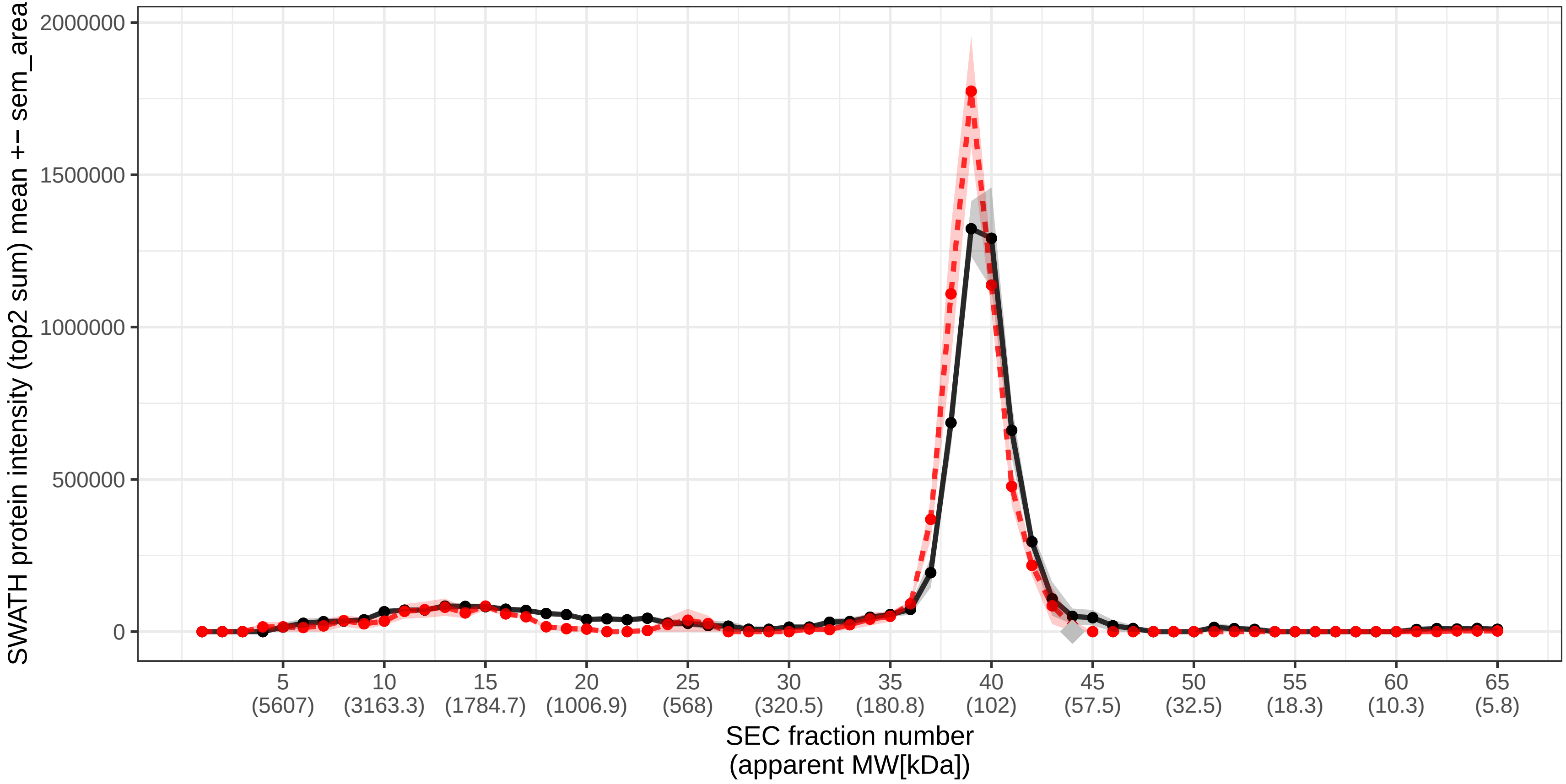

Supplement: Data S1. SEC-SWATH-MS Protein Chromatograms, Related to Figure 1 [file mmc6.zip › SECchrom_Q9H223_EHD4_HUMAN_EHD4_HCA10_HCA11_PAST4_FKSG7.pdf]
